# Outcome of the modified Dunn procedure in severe slipped capital femoral epiphysis

**DOI:** 10.1186/s13018-020-02036-3

**Published:** 2020-11-03

**Authors:** Bin Zuo, Jun Feng Zhu, Xu Yi Wang, Cheng Long Wang, Fei Ma, Xiao Dong Chen

**Affiliations:** 1 Department of Orthopaedic Surgery, Xinhua Hospital, Shanghai JiaoTong University School of Medicine (SJTUSM), Shanghai, China; 2 Department of Orthopaedic Surgery, The First Affiliated Hospital of Bengbu Medical College, Bengbu, Anhui China; 3 Shanghai Institute for Pediatric Research, Xinhua Hospital, Shanghai JiaoTong University School of Medicine (SJTUSM), Shanghai, China

**Keywords:** Slipped capital femoral epiphysis (SCFE), Modified Dunn procedure, Surgical hip dislocation (SHD), Avascular necrosis (AVN), Femoroacetabular impingement (FAI)

## Abstract

**Background:**

The modified Dunn procedure has rapidly gained popularity as a treatment for slipped capital femoral epiphysis (SCFE) during the past few years. However, there is limited information regarding its safety and efficacy in severe slips with this procedure. The purpose of this study is to present clinical results and incidence of complications associated with the modified Dunn osteotomy in a consecutive series of severe SCFE cohort.

**Patients and methods:**

We retrospectively assessed the outcomes of all twenty patients who had been treated with the modified Dunn procedure in our tertiary-care institution. According to the Loder and Fahey criteria, all cases were classified as severe slips; nineteen cases were stable, and one case was an unstable slip. All surgical procedures were performed by one senior orthopedic surgeon who had specific training in the modified Dunn procedure. Operative reports, outpatient records, follow-up radiographs, and the intraoperative findings were reviewed to determine the demographic information, type of fixation, final slip angle, presence of avascular necrosis (AVN), and any additional complications. The mean age of the patients was 13.2 ± 1.6 years (range, 10 to 17 years). Twenty patients (twenty-one hips) with a mean of 31.2 ± 14 months (range, 12 to 57 months) follow-up met the inclusion criteria. Pain and function were assessed by the modified Harris score and WOMAC score. Radiographic anatomy was measured using the slip angle and α-angle. The radiographic findings related to the anatomy of the femoral head-neck junction, as well as signs of early-onset of osteoarthritis (OA) and AVN, were evaluated pre- and postoperatively.

**Results:**

Overall, nineteen patients had excellent clinical and radiographic outcomes with respect to hip function and radiographic parameters. One patient (5%) who developed implant failure at 3 months postoperatively had a poor outcome. The mean preoperative slip angle was corrected from 63.2 ± 8.1° (range, 51 to 84°) to a normal value of 7.5 ± 3.5° (range, 2 to 15°) (*p* < 0.01). The mean α-angle was improved from an average of 94.5 ± 21.1° (range, 61 to 123°) to postoperative 42 ± 6.4° (range, 25 to 55°) (*p* < 0.01). The mean modified Harris hip and WOMAC scores postoperatively were 96.7 ± 13.4 (range, 40 to 100) and 95.4 ± 10.6 (range, 38 to 100), respectively. There were no cases of the development of femoroacetabular impingement (FAI) and the progression of OA. We did not record any case of AVN, closure of the growth plate, heterotopic ossification (HO), trochanteric nonunion, or limb length discrepancy that occurred postoperatively either at the most recent follow-up.

**Conclusions:**

Our series of severe SCFEs treated with the modified Dunn osteotomy demonstrated that the procedure is safe and capable of restoring more normal proximal femoral anatomy by maximum correction of the slip angle, minimizing probability of secondary FAI and early onset of OA. However, despite its lower surgical complication rate compared with alternative treatment described in the literature for SCFE, AVN can and do occur postoperatively which should always be concerned in every hip.

## Background

Slipped capital femoral epiphysis (SCFE) is a relatively common hip disorder presenting in adolescents with an overall incidence of about 10 cases per 100,000 children [1–3]. The main goals of SCFE treatment are to prevent further slip progression, achieve stabilization and restoration of hip function, and avoid premature hip OA while minimizing the risks of avascular necrosis (AVN) and subsequent proximal femoral deformity [4–6]. However, the management of SCFE still remains an area of controversy among orthopedic surgeons. There are conflicting reports regarding the optimal treatment and implant choice for SCFE; therefore, treatment methods must be selected on a case-by-case basis [7–9].

Historically, SCFE was usually treated with in situ fixation to prevent progression of deformity. However, SCFE is always associated with structural risk factors for hip dysfunction in addition to the risk of slip progression [9–12]. Recent studies have established an association between the residual deformity of the proximal femur after in situ treatment and the development of femoroacetabular impingement (FAI), intraarticular labrum, cartilage damage, reduced hip motion, and the progression of early-onset OA, even when the slip is mild [13–15]. Apparently, it is better to prevent these injuries than to treat it later. Nevertheless, in situ fixation alone can rarely relieve these lesions in SCFE [16, 17]. Taken together, complete restoration of normal anatomy at the slip site is the ultimate goal in terms of the aim to preserve hip motion, prevent impingement, and delay or avoid early-onset degenerations.

Realignment osteotomies have been proposed to restore the proximal femoral anatomy, but historically, results regarding AVN complication remain controversial [18]. Therefore, aiming to protect femoral head blood supply and also correct deformity, Ganz and his colleagues have recently described a modified Dunn osteotomy performed through a surgical hip dislocation (SHD) approach which could supply complete exposure of the hip joint and protection of the retinacular vessels [19, 20]. The authors stated that the technique could correct the pathoanatomy of SCFE at the level of the deformity with complete control, and the osteonecrosis rate can be theoretically decreased by meticulous dissection of the femoral neck periosteum [20–22]. Since its initial description in the literature, the modified Dunn procedure has gained popularity over the past decade in the treatment of SCFE [21, 22]. However, although many single and multicenter reports have shown that the modified Dunn procedure is a safe and effective treatment for SCFE with low rates of AVN or additional complications [4, 20, 23], limited data exists regarding its safety and efficacy, especially in severe slips.

In this study, we presented a consecutive series of severe SCFE cases adopted by the modified Dunn osteotomy with SHD approach. The primary aim of this study was to evaluate the clinical and radiographic outcomes and postoperative complication rates of patients who had undergone modified Dunn procedure. We hypothesize that this information will solidify the belief that the modified Dunn procedure is a safe surgical technique for restoring hip anatomy and function in severe SCFE.

## Methods

The study was performed at our tertiary-care institution, and was approved by the institutional review board. We retrospectively reviewed the clinical records and radiographs of a consecutive series of twenty patients (fourteen male and six female) who had undergone the modified Dunn procedure (open subcapital realignment by SHD approach) for severe SCFEs (*International Classification of Diseases, 10*^*th*^ *Revision* [ICD-10] M93.0 [slipped upper-femoral epiphysis]) from February 2015 to July 2018. Cases were restricted to codes recorded at age > 5 years and < 18 years.

Patients who underwent the in situ fixation and had the prior treatment for SCFE were excluded. The details concerning demographic information, clinical features, surgical technique, intraoperative findings, type of fixation, and postoperative complications were collected from the hospital medical records database.

Twenty patients (twenty-one hips) with a mean of 31.2 ± 14 months (range, 12 to 57 months) of follow-up whom met the inclusion criteria between February 2015 and July 2018 were reviewed. No patients were excluded because of loss to follow-up. The average age of the patients that received operation was 13.2 ± 1.6 years (range, 10 to 17 years). Five hips were involved in the left side and fourteen hips in the right side. Besides, one female patient was noted severe SCFE in the contralateral side. Preoperatively, the slips were described as stable or unstable based on the clinical history at the time of presentation according to the criteria of Loder et al. [24]. Onset of symptoms was also recorded, and slips were classified as acute, chronic, and acute-on-chronic according to the Fahey and O’Brien classification system [25]. Table 1 shows the demographic characteristics of this series.

**Table 1:** Demographics and preoperative SCFE characteristics

Patients (no. of hips)	20 (21)
Age (range) (year)	13.2 ± 1.6 (10–17)
Sex (F:M)	14:06
Hip (L:R)	6:15
Classification (no. of hips)	
Loder (U:S)	1:20
Fahey/O’Brien (acute:chronic)	1:20
Duration of follow-up (range) (months)	31.2 ± 14 (12–57)
Slip angle (°)	63.2 ± 8.1 (51–84)
α angle (°)	94.5 ± 21.1 (61–123)

*M* male, *F* female, *L* left, *R* right, *U* unstable, *S* stable

**Fig. 1:**
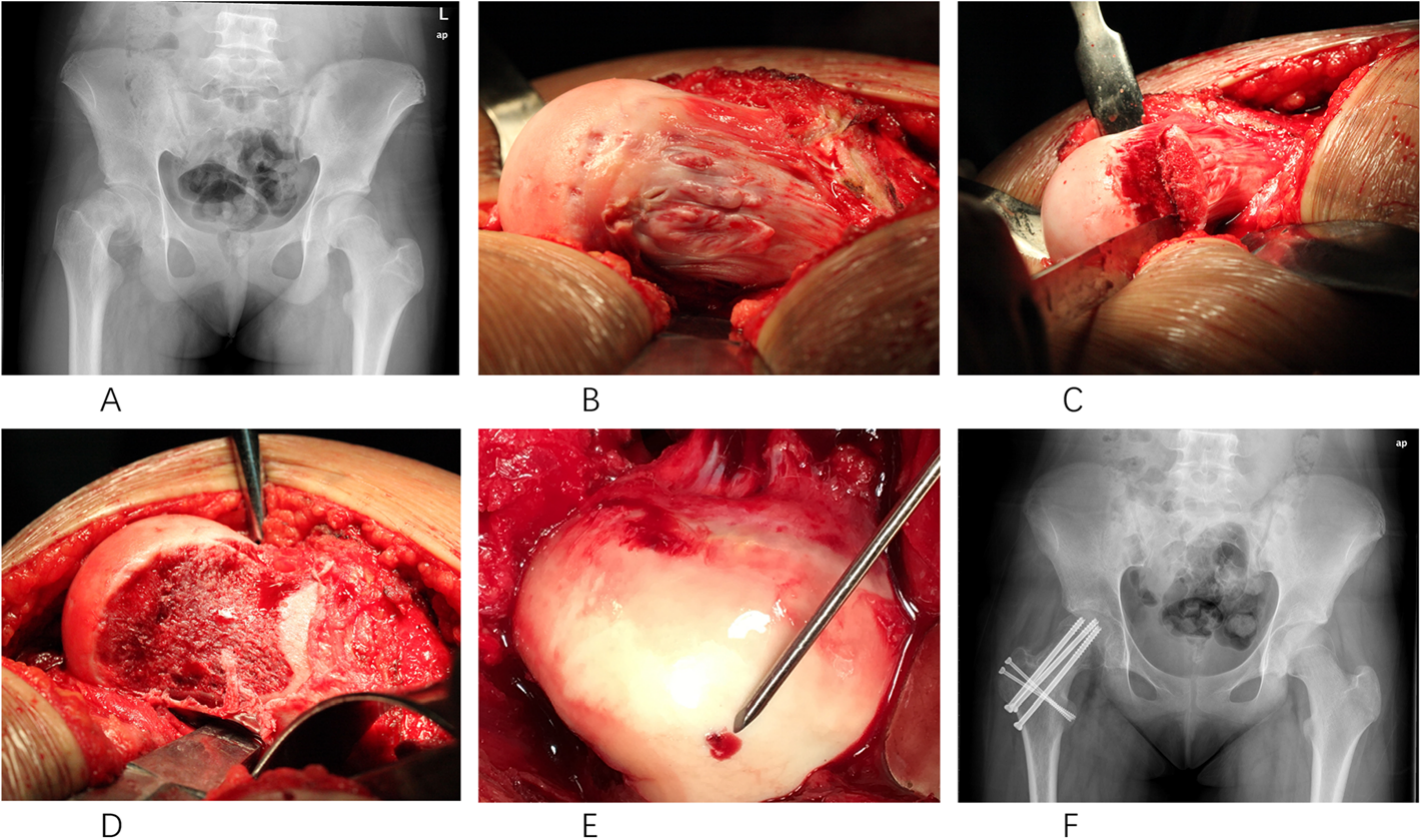
A 12-year-old female patient presented with pain in the right hip. **a** Anteroposterior (AP) radiograph demonstrates severe posteromedial displacement of the capital femoral epiphysis. **b** Intraoperative picture shows the displacement. **c** Modified Dunn osteotomy after surgical hip dislocation (SHD). **d** Femoral osteochondroplasty was performed to restore the proximal femoral anatomy and femoral head sphericity. **e** Positive bleeding sign after reduction. **f** Postoperative AP view at 1-year follow-up with excellent hip motion

**Fig. 2:**
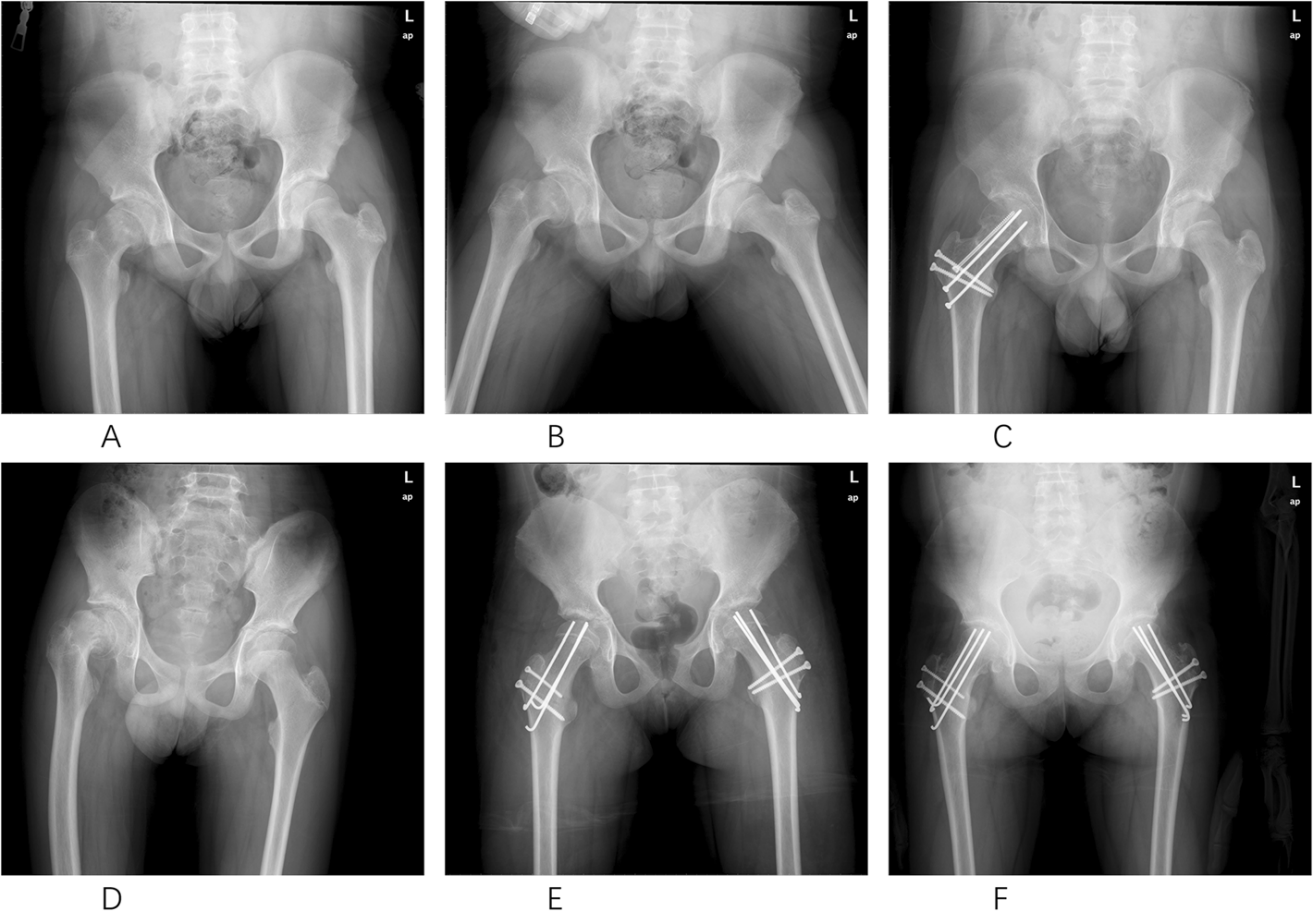
**a**–**c** A 10-year-old girl with severe pain and functional disability. **a** AP and **b** frog-leg lateral radiographs revealing a severe SCFE of the right hip. **c** AP radiographs obtained 2 years after surgery with no signs of AVN. AP indicates anteroposterior. **d**–**f** A 17-year-old girl with bilateral severe SCFE. **d** AP radiographs revealing severe SCFE of the bilateral hips. **e** AP radiographs obtained after the fixation. **f** AP radiographs obtained 2 years after surgery with no signs of AVN. AP indicates anteroposterior

All patients were treated with the modified Dunn procedure as described by Ganz et al. [20] by one senior surgeon (Chen XD) who had specific training in the modified Dunn procedure and surgical hip dislocation approach (Fig. 1a–f). Table 2 shows intraoperative findings, and the type of fixation varied according to intraoperative findings including threaded Kirschner wires (4 hips), 4.5-mm screws (5 hips), 6.5-mm cannulated screws (12 hips), and 2.8 Suture Anchor system (TWINFIX Ti 2.8 Suture Anchor w/ONE 38” ULTRABRAID #2 Suture, Endoscopy Smith & Nephew Inc, Andover, USA) (e.g., if intraarticular labral tear coexisting) (5 hips) (Fig. 2). Intraoperative monitoring of femoral epiphyseal perfusion was performed by drilling a 2-mm hole in the anterior femoral head to assess for active bleeding [26] (Fig. 1e).

**Table 2:** Intraoperative findings

Intraoperative finding	Patients (no. of hips)
Cartilage damage	
Evidence of damage	20
No damage	1
Labral damage	
Evidence of damage	5
No damage	16
Femoral head-neck junction deformity	
Evidence of deformity	21
No deformity	0
Fixation types	
K-Wires	4
Screws	17
Suture anchor	5
Bleeding of the femoral head after the reduction	
Bleeding	21
No bleeding	0

With surgical dislocation, a full 360° view of the femoral head can be exposed, and the femoral head-neck junction, labrum, and acetabular cartilage are simultaneously evaluated for the presence of any lesions. The minimal amount of bone necessary is resected to restore femoral head sphericity if any nonspherical portions of the femoral head exist (Fig. 1d). The labrum is reattached with bone anchors placed around the acetabular rim, approximately 1.0 to 1.5 cm apart, close to the subchondral plate if any labral tears exist. The capsule must be closed without tension (to avoid compromising the blood supply to the femoral head) at the site of arthrotomy. Debridement of degenerated labrum and/or cartilage and femoral osteochondroplasty (Fig. 1d) was always performed as most of the patients had morphological abnormalities upon intraoperative finding. Assessment of intraoperative impingement was repeated after each resection step to avoid residual FAI and to minimize the amount of resection.

Preoperative and postoperative slip angles were documented according to Southwick method [27]. Follow-up images including supine anteroposterior and frog-leg lateral radiographs were reviewed by two senior surgeons (Dr. ZH JF and Dr. Z B) to determine the presence of osteonecrosis (collapse or sclerosis of the femoral head), FAI, the Tönnis grade of osteoarthritis [28], and any additional complications. α-angles at last follow-up were also analyzed [29].

Patients were placed on a restricted non-weight bearing protocol for 6 to 8 weeks postoperatively, followed by protected weight bearing with crutches for additional 4 to 8 weeks, depending on radiographic healing status. Physical therapy involving gait training and strengthening was then initiated, and vigorous activity was generally restricted for 6 months postoperatively.

### Statistical analysis

Continuous variables were tested for normality and are reported as mean ± SD. Paired Student’s *t* tests were used to determine the significance of patient improvements in outcome scores and changes in slip angle and α-angle. All tests were 2-sided, and a *p* value of < 0.05 was considered significant. Descriptive statistics were calculated using Statistical Package for Social Sciences (SPSS) (Version 24.0, IBM, Armonk, NY, USA).

## Results

According to the Loder and Fahey criteria, nineteen cases were stable, and one case was unstable slip; nineteen cases were chronic, and one case was acute slip. With the use of the slip angle classification, all slips were classified as severe slips (slip angle > 50°) (Table 1).

All patients underwent osteochondroplasty of the femoral head-neck junction and labral tear detection simultaneously. Five hips had labral tears that required repair with two or three 2.8-mm suture anchor. No patients received prophylactic pinning with an unaffected contralateral hip. Intraoperative monitoring of femoral epiphyseal perfusion was performed by peripheral drilling of a 2-mm hole with K-wire in the anterior femoral head to assess for active bleeding; all heads were positive bleeding before dislocation. After epiphyseal reduction, peripheral K-wire drilling was repeated to avoid tension on posterior retinaculum; all femoral heads were positive bleeding. Only one hip developed implant failure postoperatively with a positive “bleeding sign”. Table 2 shows the intraoperative findings.

The mean follow-up was 31.2 months (range, 12 to 57 months). The mean slip angle was corrected from preoperative 63° ± 8.1° (range, 51 to 84°) to postoperative 7.5° ± 3.5° (range, 2 to 15°) (*p* < 0.01). The mean α-angle improved from a preoperative value of 94.5° ± 21.1° (range, 61 to 123°) to postoperative 42° ± 6.4°(range, 25 to 55°) (*p* < 0.01).

As regards the mean postoperative HHS in our series, it was 96.7 ± 13.4 (range, 40 to 100), and the mean WOMAC score was 95.4 ± 10.6 (range, 38 to 100) [30, 31]. According to the two criteria, 19/20 patients (20/21 hips) acquired excellent clinical results (Table 3).

**Table 3:** Radiographic and clinical findings

Slip angle (°)	7.5 ± 3.5 (2 to 15)
α angle (°)	42 ± 6.4 (25 to 55)
Tönnis grade of OA (no. of hips)	
0	20
1	0
2	0
3	1
HHS score (range)	96.7 ± 13.4 (40 to 100)
WOMAC score (range)	95.4 ± 10.6 (38 to 100)
Complications (no. of hips)	
AVN	0
FAI	0
Trochanteric nonunion	0
Infection	0
Implant failure	1

No statistically significant differences were found between groups regarding sex distribution, age at presentation, BMI and BMI-for-age percentile, time elapsed from initial symptoms, and preoperative PSA.

**Fig. 3:**
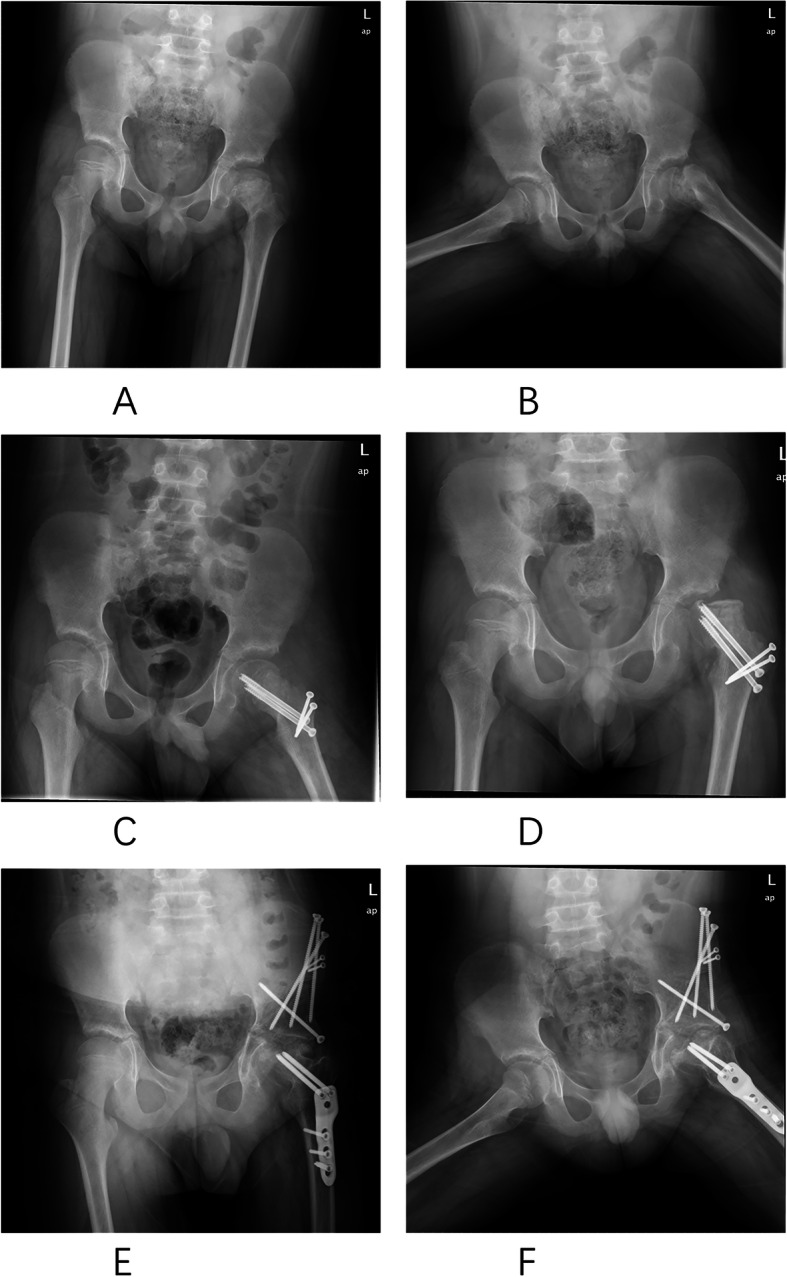
A 10-year-old boy with severe pain and functional disability. **a** AP and **b** frog-leg lateral radiographs revealing a severe SCFE of the left hip. **c** AP showed good restoration of the SCFE after the operation. **d** Obtained 3 months postoperatively showed implant failure. And finally received periacetabular osteotomy and proximal femur osteotomy surgery, **e** AP and **f** frog-leg lateral radiographs. AP indicates anteroposterior

Postoperatively, one patient (5%) developed implant failure within 3 months of osteotomy which was thought to be due to non-compliance with weight-bearing restrictions. This patient had originally been stabilized with two 4.5-mm solid screws and received revision operation with periacetabular osteotomy (PAO). There was some heterotopic ossification of the left hip at the last follow-up, and the hip function and OA progression required further follow-up in this case (Fig. 3). The remaining patients healed uneventfully after primary fixation. There were no cases of fixation failure involving the trochanteric osteotomy. We did not record any case of AVN, early onset of OA, symptomatic FAI, trochanteric nonunion, or HO that occurred postoperatively. No closure of the growth plate was observed, and no limb length discrepancy was recorded at 12 months postoperative follow-up in the cohort. There were no other complications, including infection or neurologic injury in the series. Overall, one implant failure complication occurred in the twenty patients (an overall rate of 5%).

## Discussion

Treatment of severe SCFE remains a challenging problem. Traditionally the goal of primary treatment of SCFE has been to stabilize the epiphysis and prevent additional displacement and complications, thereby restoring reasonable function and delaying or preventing OA [11].

In situ fixation in the treatment of SCFE had been traditionally defined as a safe and reliable method in mild SCFEs [10, 11]. The use of this surgical technique with a modified screw allows residual growth of the femoral neck following mild SCFE and permits restoration of the anatomy of the proximal femur while avoiding development of FAI following mild SCFE [32]. However, recent evidence suggests that this procedure does not address the posterior tilt and rotation, or translation of the epiphysis in severe cases [7]. Therefore, the hip may remodel into an abnormal femoral head-neck junction and the residual deformities potentially leading to FAI and subsequent early-onset OA in those severe SCFEs [1, 4, 7, 9]. In addition, an investigation in cadaveric human femora reported approximately 70% incidence of a severe degree of OA in hips with minimum postslip morphology [29]. Obviously, the widely accepted in situ fixation might not be adequate to accomplish these goals. Managing the SCFE-triggered epi-/metaphyseal deformities by femoral or acetabular realignment has emerged as an intractable issue that needs to be addressed [6]. In conclusion, an ideal treatment strategy of SCFE should aim to restore the original anatomy to avoid subsequent early hip degeneration, pain, and the need for THA in young and active patients with SCFE [20].

To resolve the above issues, the modified Dunn procedure has been reported to be a promising technique which can address both physeal stability and residual deformity with potential lower complication rates in the treatment of SCFE [20]. Concurrently, intra-articular pathology such as articular cartilage damage and labral tear can also be addressed via this approach.

Several studies have reported on the treatment of both stable and unstable SCFE with SHD and the modified Dunn procedure [20–23]. In the most recent update, there were 21 excellent results and only 2 poor results because of AVN (9.5%) [18]. Huber et al. [22] reported a separate cohort treated with SHD and a modified Dunn operation. Twenty-eight of 30 hips had excellent results, and only 1 hip had a poor result because of AVN.

The most worrisome complication of SCFE treatment is AVN which is recognized as a major risk factor for early development of OA and hip replacement [20]. However, as regards the modified Dunn osteotomy with SHD, the incidence of complications was low. In Huber et al.’s study, they recorded one case of AVN (3.5%) [22]; in Massè et al.’s study, they did not record any case of AVN [33]; in Slongo et al.’s study, they also recorded one case of AVN (4.4%) [21]. In our study, there was no case developed to postoperative AVN.

FAI has been associated with increased pain, reduced ROM, chondrolabral damage, and early hip osteoarthritis [20, 21]. Residual abnormal morphology of the proximal femur is currently believed to be the mechanical cause of FAI and early articular cartilage damage in SCFE [13–15]. Recent studies investigating clinical and radiographic evidence of FAI during the first decade after SCFE treatment revealed a persistent femoral deformity in patients undergoing in situ fixation [13, 21]. Dodds et al. reported pain in 31% of 49 patients with a mean follow-up of 6.1 years after in situ pinning [34]. Fraitzl et al. reported on 16 patients who underwent in situ pinning for mild SCFE with an average of 14 years of follow-up [35]. None of the 16 patients had normal proximal femoral morphology assessed by the head-neck offset ratio. The modified Dunn osteotomy had been indicated to realign the proximal femur especially in moderate to severe SCFE [20–23]. In the current series, we recorded no FAI and OA progression postoperatively.

Similar to the causes of FAI, the repetitive trauma from impingement can also lead to intra-articular pathology such as labral tears and injury to the articular cartilage [20]. Acetabular cartilage and labral injury after SCFE have been widely reported both at initial presentation and during deformity correction even in mild slips [21]. Despite the fact that the metaphysis undergoing some remodeling over time, the residual SCFE deformity can still cause hip pain, functional impairment, and progression to joint degeneration due to impingement of the anterosuperior deformity with the acetabulum [17, 24]. Leunig et al. reported universal hip pathology at the time of fixation of the SCFE, through both open surgery and arthroscopy [14]. The authors postulated that mechanical jamming was the main factor causing direct and early acetabular rim and cartilage damage that may lead to hip OA. In a retrospective study, Sink et al. [36] reported acetabular cartilage injury in 33 and labral injury in 34 of 39 hips at the time of surgical dislocation of the hip for the treatment of symptomatic stable SCFE. Similarly, Ziebarth et al. found a high incidence of acetabular cartilage and labral lesions visualized at the time of surgical dislocation [20]. The modified Dunn procedure through SHD could expose the acetabular well allowing maximum reduction of slips and concomitant osteochondroplasty for correction of residual femoral neck deformity. Our study found that patients who underwent a modified Dunn procedure also had improved pain and function at a medium-term follow-up.

The modified Dunn is technically demanding, and the issue of the steep learning curve has been highlighted in previous studies [2–4]. Upasani et al. [37] reported an important association between the surgeon’s volume and experience in performing the procedure and the likelihood of major complications. In our series, the rate of AVN was superior than previous reports of surgeons at the beginning of the learning curve [4, 37, 38].

The failure case in our cohort showed in Fig. 3 is controversial to some extent. Preoperative imaging findings combined with CT and X-ray showed suspicious necrosis of the femoral head; however, the weight-bearing surface was acceptable, and the femoral head was positive bleeding before dislocation during the operation. Additionally, the treatment of femoral head necrosis in adolescents is quite different from that in adults which prognosis used to be better. We therefore decided to try the hip preservation treatment in consideration of the above reasons. This failure case indicates that the failure rate of similar cases is much higher. There are few relevant literature reports, and the similar cases need to be further explored.

Our study has several limitations. The retrospective design and lack of randomization introduced potential selection biases. Without a truly randomized long-term study about results of SHD combined with modified Dunn osteotomy, it would be difficult to compare outcomes of this technique with those of various other surgical techniques such as in situ pinning, capital realignment, and intertrochanteric osteotomy. Another major limitation in our study is lack of long-term follow-up in comparison with other studies. Longer follow-up will be necessary to assess the long-term success of the procedure. Besides, a small number of patients and short-term follow-up do affect the information we could interpret. As this report includes the duration of 3-year cases, the intraoperative and postoperative protocols were not very uniform. The type of fixation varied widely among the cases, and the small numbers of patients treated with each type of implant limited our ability to draw definitive conclusions regarding the optimal type of fixation. In addition, the intraoperative monitoring of epiphyseal perfusion was dependent on rough method (peripheral K-wire drilling), which prevented us from sensitively assessing potential changes in blood flow over the course of the procedure. Doppler ultrasound probe can detect perfusion of both epiphysis and retinaculum so can be a good reliable method for predicting the development of postoperative AVN. In addition to plain radiography, we also perform CT or MRI in some patients to detect the incidence of osteonecrosis of the epiphysis or penetration of the fixation screws, but not all of the cases due to the medical costs. MRI and CT can reflect the early osteonecrosis well, and standardized imaging can also make sure that inter-study comparison is possible. We plan to adopt CT and MRI as routine examinations in subsequent cases. Nevertheless, the role of modified Dunn osteotomy via SHD approach to correct severe SCFE deformity and avoid FAI-related cartilage damage and development of OA has been conveyed in our study.

## Conclusion

In spite of its limitations, this study represents a series of severe SCFE cases treated with the modified Dunn procedure via SHD approach. Our series of severe SCFEs treated with the modified Dunn osteotomy demonstrated that the procedure is safe and extremely valuable for restoring hip anatomy and preserving function after a severe slip. This procedure is technically demanding, and the safe execution requires full understanding of the vascular anatomy of the hip.

## Data Availability

The dataset supporting the conclusions of this article is included within the article.

## Abbreviations

SCFE: Slipped capital femoral epiphysis
AVN: Avascular necrosis
OA: Osteoarthritis
FAI: Femoroacetabular impingement
HO: Heterotopic ossification
SHD: Surgical hip dislocation
SPSS: Statistical Package for Social Sciences
PAO: Periacetabular osteotomy
